# Spatiotemporal and Kinematic Parameters Relating to Oriented Gait and Turn Performance in Patients with Chronic Stroke

**DOI:** 10.1371/journal.pone.0129821

**Published:** 2015-06-19

**Authors:** Céline Bonnyaud, Didier Pradon, Nicolas Vuillerme, Djamel Bensmail, Nicolas Roche

**Affiliations:** 1 Inserm Unit 1179, Team 3: Technologies and Innovative Therapies Applied to Neuromuscular diseases, UVSQ, CIC 805, APHP Service de physiologie et d’exploration fonctionnelle, Hôpital Raymond Poincaré, 92380, Garches, France; 2 Inserm Unit 1179, Team 3: Technologies and Innovative Therapies Applied to Neuromuscular diseases, UVSQ, CIC 805, APHP Service de Médecine Physique et Réadaptation, Hôpital R. Poincaré, AP-HP, Garches, France; 3 Univ. Grenoble Alpes Laboratoire AGIM, La Tronche, France; 4 Institut Universitaire de France, Paris, France; Centre de Psychiatrie et Neurosciences, Hopital Sainte-Anne and Université Paris, FRANCE

## Abstract

**Background:**

The timed up and go test (TUG) is a functional test which is increasingly used to evaluate patients with stroke. The outcome measured is usually global TUG performance-time. Assessment of spatiotemporal and kinematic parameters during the Oriented gait and Turn sub-tasks of the TUG would provide a better understanding of the mechanisms underlying patients’ performance and therefore may help to guide rehabilitation. The aim of this study was thus to determine the spatiotemporal and kinematic parameters which were most related to the walking and turning sub-tasks of TUG performance in stroke patients.

**Methods:**

29 stroke patients carried out the TUG test which was recorded using an optoelectronic system in two conditions: spontaneous and standardized condition (standardized foot position and instructed to turn towards the paretic side). They also underwent a clinical assessment. Stepwise regression was used to determine the parameters most related to Oriented gait and Turn sub-tasks. Relationships between explanatory parameters of Oriented gait and Turn performance and clinical scales were evaluated using Spearman correlations.

**Results:**

Step length and cadence explained 82% to 95% of the variance for the walking sub-tasks in both conditions. Percentage single support phase and contralateral swing phase (depending on the condition) respectively explained 27% and 56% of the variance during the turning sub-task in the spontaneous and standardized conditions.

**Discussion and Conclusion:**

Step length, cadence, percentage of paretic single support phase and non-paretic swing phase, as well as dynamic stability were the main parameters related to TUG performance and they should be targeted in rehabilitation.

## Introduction

Patients with stroke-related hemiparesis frequently have impaired balance and gait, limiting daily life activities. The improvement of locomotor skills is therefore a major aim of stroke rehabilitation [1] and an accurate assessment of the patient’s impairments and function is essential for treatment planning (surgical, pharmacological or physiotherapy-related). The Timed Up and Go (TUG) test [2] is widely used to assess locomotor capacity in stroke patients [3]. This test measures the time required to rise from a chair, walk 3 meters, turn, walk back and sit down again, thus evaluating tasks which are regularly encountered in daily life. Although the TUG is a good general indicator of locomotor function, the timed global performance does not provide any information regarding the mechanisms underlying the patient’s disabilities and specific problems relating to each sub-task are not highlighted [4]. Wall et al (2000) thus proposed the Expanded Timed Up and Go test, using video recordings of each sub-task in order to identify the impairments which reduce the patient’s performance [4]. Similarly, Faria et al (2013) proposed the TUG-ABS (Assessement of Biomechanical Strategies) in order to aid decision making. It consists of a 15-item scale of biomechanical strategies for each sub-task of the TUG [5]. The purpose of both these tests is to identify the mechanisms which reduce patient performance in each sub-task of the TUG.

Motion analysis would be a pertinent method to investigate biomechanical aspects of the TUG. The use of instrumental biomechanical tools to assess functional tasks has increased over the past few years. Galli et al (2008) and Lecours et al (2008) both quantified kinematics and kinetics during sit to stand in subjects with stroke and healthy subjects [6, 7]. Dion et al (2003) and Frykberg et al (2009) assessed a sit to walk task in stroke patients using a 3D optoelectronic system and force plates [8, 9]. Several studies have evaluated the TUG test using accelerometers in patients with Parkinson’s disease and healthy subjects [10, 11, 12]. The pertinence of the accelerometers was demonstrated by the fact that the timed TUG performance did not differentiate between the groups but the accelerometer analysis did. Range of motion during sit-to-stand and stand-to-sit, turning velocity, cadence and trunk rotation velocity were all found to be reduced in the patients [10, 11].

Three-dimensional analysis using an optoelectronic system is the current gold standard for the biomechanical assessment of patients with gait abnormalities [13]. This method is pertinent for the analysis of spatio-temporal and kinematic parameters of the paretic and non-paretic lower limbs during each sub-task of the TUG and would increase understanding of the main mechanisms which underlie performance in stroke patients. Moreover, the results would help to optimize rehabilitation techniques which aim to improve locomotor capacity.

The aim of this study was thus to determine which spatio-temporal and/or kinematic parameters would be the most related to performance in Oriented gait and Turn sub-tasks of the TUG test (time to perform the sub-task) in stroke patients. We hypothesized that the percentage of single support phase and peak hip extension on the paretic side would be particularly related to the performance of Oriented gait and Turn sub-tasks of the TUG. The percentage of single support phase during gait has been shown to predict the time to perform the entire TUG test and peak hip extension has been shown to be associated with gait speed [14, 15].

## Methods

### Subjects

Twenty nine participants with chronic hemiparesis were included (18 men and 11 women, mean age 54.2±12.2 years) (Table 1). The inclusion criteria were: age over 18 years, hemiparesis due to stroke, ability to carry out the TUG test several times without any assistive devices and medically stable enough for participation in the protocol. Patients were excluded if they had other neurological, orthopedic or medical disorders that might interfere with the test. All subjects gave written consent before participation. This study was performed in accordance with the ethical codes of the World Medical Association, was approved by the local ethics committee (Comité de protection des personnes Ile de France XI, Ref 13005. CNIL, Ref DR-2013-283) and the individuals have given their written informed consent.

**Table 1:** Participants characteristics.

Subjects	Sex (m/w)	Age (Years)	Height (m)	Weight (kg)	Hemiparetic side	Duration of the lesion (years)	Spasticity (Sum)	Claw toe (yes/no)	MRC (Sum)	Foot sole pressure	Toe proprioception	BBS	ABC	Fall frequency	Fear of falling
1	m	53	1.76	69	right	10	5	y	24	1	2	46	64.4	0	5
2	m	60	1.68	71	right	6	6	y	13	2	3	50	86.3	0	2
3	m	72	1.58	62	left	10	3	y	24	2	3	51	51.3	0	10
4	w	46	1.57	70	left	18	8	y	23	2	1	51	64.4	1	5
5	m	63	1.76	92	right	29	1	y	21	1	0	50	68.1	0	6
6	w	67	1.52	60	left	9	2	n	33	2	3	54	78.8	0	0
7	w	71	1.68	58	left	11	5	y	19	2	3	45	71.3	1	8
8	m	56	1.67	69	left	3	2	n	30	2	3	54	73.8	1	0
9	m	45	1.72	108	left	9	9	y	20	1	1	47	80.0	1	0
10	m	52	1.7	80	right	1	7	n	20	1	2	50	75.9	2	2
11	m	43	1.65	68	right	8	0	n	18	2	3	52	82.5	0	5
12	m	33	1.7	68.5	left	5	7	y	24	2	3	52	88.1	1	1
13	m	33	1.75	87	right	2	13	y	13	1	2	51	86.9	1	0
14	m	61	1.83	83	left	9	4	y	23	2	3	49	56.9	1	5
15	m	57	1.87	101	left	5	11	n	17	1	3	49	91.9	1	1
16	m	52	1.76	105	right	8	1	n	29	1	0	54	95.6	1	0
17	m	59	1.6	85	left	12	9	y	10	1	1	52	93.1	0	0
18	m	58	1.8	85	right	1	9	y	13	1	2	50	86.3	0	1
19	m	60	1.76	68	left	11	1	y	18	2	3	51	81.9	0	2
20	w	44	1.68	60	right	14	1	y	19	1	1	51	65.0	1	0
21	w	40	1.62	55	right	3	3	y	25	1	0	52	79.4	0	2
22	w	47	1.6	50	right	5	0	y	23	1	0	50	46.9	0	9
23	w	67	1.55	48	left	8	3	n	24	1	3	49	70.6	1	1
24	w	27	1.6	60	right	5	4	n	32	2	1	54	94.4	2	3
25	m	47	1.77	66	left	5	10	n	27	1	0	50	90.0	0	1
26	w	66	1.65	80	left	0.5	4	y	25	2	3	51	85.0	1	5
27	m	75	1.78	89	left	7	1	y	27	1	2	48	65.6	2	5
28	w	64	1.62	60	left	8	3	n	21	2	2	49	71.3	1	10
29	w	54	1.59	63	left	7	6	n	18	2	3	53	66.9	0	2

Spasticity: sum of quadriceps, rectus femoris, hamstring and triceps surae assessed with Modified Ashworth Scale (0–4). MRC (Medical Research Council scale): sum of hip, knee and ankle flexors and extensors strength. Foot sole pressure was assessed with the Nottingham Sensory Assessment (0 = absent, 1 = impaired, 2 = normal). Toe proprioception was assessed with the Nottingham Sensory Assessment (0 = absent, 1 = direction incorrect, 2 = direction ok, inaccurate position, 3 = direction ok, position accurate to 10°). BBS: Berg Balance Scale (0 to 56). ABC: Activities-specific Balance Confidence (0 to 100%). Fall frequency: number of falling within 3 last months. Fear of falling between 0 (no fear) and 10 (extreme fear of falling).

### Experimental procedure

#### TUG test analysis: Data collection and processing

3D-TUG analysis was carried out using an optoelectronic motion capture system (sampling frequency 100 Hz, Motion Analysis Corporation, Santa Rosa, CA, USA). Markers were fixed to specific bony landmarks on both sides of the body according to the Helen Hayes marker set [16, 17]: the middle-toe, the heel, the medial and lateral malleoli of the ankle, the shank, the medial and lateral femoral condyles, the thigh, the anterior superior iliac spines, the tip of the acromion process, the lateral epicondyle of the humerus, the center between the styloid processes of the radius and ulna, the sacrum and an offset was fixed over the right scapulae. The greater trochanter and the anterior superior iliac spine were added to improve the reconstruction of the trajectories of joint coordinate systems. To ensure good reliability, the same person positioned all the markers on all the subjects [13] and participants all wore the same type of comfortable shoes [18]. Participants were seated on a stool with their arms held out from the body [19, 9]. They were asked to stand up, walk 3m to a cone, turn around the cone, return to the stool and sit down, at their natural speed without any walking aids or orthoses. Three trials were recorded for each condition (described below).

It has been shown that seat height, foot position and turning direction influence the sit to stand movements and TUG performance in healthy and stroke subjects [20, 21, 22, 23]. Some studies have attempted to simulate real life conditions during the TUG (standard chair height, natural starting position) [9, 22] while others have used standardized conditions [8, 21, 22]. In the present study, Oriented gait and Turn sub-tasks performance were assessed in both the spontaneous (Spont) and standardized (Stand) conditions. The Spont condition was performed first [22]. In this condition, subjects sat on a 45cm-high stool to imitate standard chair height [9], they could position their feet freely and no instruction was given regarding the direction of the turn. In the standardized condition (Stand), seat height was set to 100% of the distance from the fibular head to the floor [20], knees were flexed at 100° and feet were placed symmetrically [24, 21]. Participants were instructed to look at the cone at the beginning of the task and to turn towards the paretic side.

Marker trajectories were recorded using 8 infrared cameras and filtered using a low-pass Butterworth filter with a cut off frequency of 6 Hz [25]. Anatomical frames were defined from the position of the markers in the reference standing position. This model was used to analyses the spatio-temporal and kinematic parameters. Open-source Biomechanical Tool Kit package for MATLAB [26] was used to define the phases of the gait cycle and sub-tasks of the TUG. The gait phases were defined according to Perry [27] and sub-tasks of the TUG were defined according to previous studies [9, 28, 29]. Three sub-tasks were analyzed i) ‘Go’ = walk forward to cone: begins at toe off of the first step and ends with the first foot strike in the direction of the turn; ii) ‘Turn’ = walk around the cone: ends at the first foot strike lined up with the stool [28] and iii) Return = walk back to stool: ends with foot strike of the last step prior to the turn to sit. The decision not to analyze the two other sub-tasks of the TUG (stand-up and sit down are discussed in the limits section).

The data were then exported to Matlab (R14, The MathWorks Inc., Natick, MA, USA) for calculation of the biomechanical parameters in each sub-task.

The parameters analyzed were:

1. Time taken to perform each sub-task, which corresponded to TUG performance.
2. Spatiotemporal parameters: cadence, width, and step length and percentage of single support phase (%SSP) and swing phase (%SP) for each limb.
3. Kinematic parameters: peak flexion and extension of the hip, knee and ankle on the paretic and non-paretic sides. For the ankle, maximal dorsiflexion was also calculated during swing phase.

#### Clinical evaluation

Spasticity of the whole quadriceps, rectus femoris (one head of the quadriceps), hamstring and triceps surae was evaluated with the Modified Ashworth Scale (MAS) [30]. Strength of the hip, knee and ankle flexor and extensor muscles was assessed with the Medical Research Council (MRC) scale [31]. The scores of the MRC and MAS were summed. The presence of claw toes was also noted and sensory impairment was assessed with the Nottingham Sensory Assessment [32]. The Berg Balance Scale (BBS) was used to evaluate balance capacity [33, 34] and the Activities-specific Balance Confidence (ABC) scale was used to quantify the level of confidence (from 0 to 100%) to carry out activities without losing balance [35]. Participants were also asked to report the number of falls within the last 3 months and to estimate their fear of falling on a visual analog scale between 0 (not afraid) and 10 (extreme fear of falling). The same physiotherapist assessed all the participants.

### Statistical analysis

Descriptive statistics including means and standard deviations were calculated for each parameter and Oriented gait (Go, Return) and Turn in both conditions (Spont and Stand). To identify the spatiotemporal and kinematic parameters which were the most related to Oriented gait (Go and Return) and Turn performance, a stepwise multiple regression analysis with forward selection was used. The number of variables included in the stepwise analysis has to be small compared to the number of subjects [36]. Firstly, to select the data entered in the stepwise model, we performed Pearson’s correlations between all spatiotemporal and kinematic parameters and Go, Turn and Return performance (level of significance p<0.05). Spatio-temporal and kinematic variables which were significantly correlated with TUG performance were then used for the stepwise analysis. The stepwise multiple regression is particularly recommended to assess the association between several independent variables and a single continuous variable. It selects parameters that best explain the variability of TUG at a significance level of p<0.01 [37, 38]. Multiple linear regression analysis is an extension of simple linear regression used to assess the association between two or more independent variables and a single continuous variable. The results of a multiple linear regression is expressed by the following equation: Y = b_0_+ b_1_X_1_+ b_2_X_2_+….+ b_p_X_p_ where Y is the explanatory value, X_1_ through X_p_ are p distinct explanatory variables, b_0_ is the value of Y when all the independent variables (X_1_ through X_p_) are equal to zero and b_1_ to b_p_ are the estimated regression coefficients. Each regression coefficient represents the change in Y relative to a one unit change in the respective independent variable.

A Spearman’s test was then used to evaluate correlations between variables found to explain TUG performance from the stepwise analysis and clinical tests since clinical data were not continuous (level of significance p<0.05).

## Results

Results of the Oriented gait (Go and Return) and Turn performance and spatiotemporal and kinematic parameters for each sub-task and both conditions (Spont and Stand) are presented in Table 2. Mean (sd) time to perform the task was:

**Table 2:** TUG performance and spatiotemporal and kinematic parameters for each sub-task and both conditions (Spont and Stand).

	Spont			Stand		
	Go	Turn	Return	Go	Turn	Return
TUG performance (s)	4.83 (1.18)	2.98 (0.73)	4.23 (1.02)	4.56 (1.01)	3.16 (0.84)	3.81 (0.91)
Cadence (step/min)	92.27 (10.98)	92.98 (15.36)	91.3 (10.52)	93.48 (11.13)	92.6 (11.82)	92.99 (10.49)
Width (cm)	16.14 (5.19)	17.63 (6.14)	15.86 (5.07)	17.10 (5.38)	22.33 (4.75)	15.99 (4.82)
Step length paretic side (cm)	45.51 (8.03)	31.38 (10.09)	42.73 (7.66)	45.29 (8.15)	27.69 (9.82)	43.92 (7.09)
Step length non paretic side (cm)	40.58 (10.23)	27.08 (11.14)	41.34 (9.61)	42.29 (8.9)	31.7 (9.21)	42.69 (8.8)
% SSP paretic side (%)	28.09 (3.87)	25.26 (4.99)	28.65 (3.75)	28.45 (3.98)	26.8 (4.3)	29.2 (3.69)
% SSP non paretic side (%)	39.56 (3.71)	38.39 (3.29)	39.2 (3.08)	39.9 (3.36)	36.52 (4.35)	39.15 (2.83)
% SP paretic side (%)	39.14 (3.49)	38.19 (3.13)	38.48 (3.08)	39.34 (3.28)	36.53 (4.13)	38.63 (2.87)
% SP non paretic side (%)	28.54 (3.76)	24.67 (4.93)	28.53 (3.70)	28.65 (3.62)	26.78 (4.3)	29.34 (3.53)
Peak hip flexion paretic side (°)	41.79 (10.27)	37.63 (9.33)	37.36 (9.44)	40.57 (10.59)	35.93 (9.64)	36.42 (9.6)
Peak hip flexion non paretic side (°)	47.42 (8.42)	43.09 (7.64)	45.53 (8.15)	47.15 (8.42)	43.77 (8.25)	44.83 (8.13)
Peak hip extension paretic side (°)	-2.65 (8.58)	-6.06 (9.22)	-1.57 (8.42)	-2.83 (8.54)	5.47 (9.34)	-1.15 (8.32)
Peak hip extension non paretic side (°)	4.32 (8.23)	-0.48 (8.44)	5.03 (8.32)	4.45 (8.77)	3.06 (8.61)	5.63 (8.66)
Peak knee flexion paretic side (°)	45.31 (8.7)	41.35 (9.53)	42.93 (10.74)	44.13 (8.58)	40.15 (8.43)	44.28 (10.36)
Peak knee flexion non paretic side (°)	70.33 (5.25)	66.07 (8.17)	70.27 (5.33)	70.49 (5.03)	69.41 (5.61)	69.93 (5.14)
Peak knee extension paretic side (°)	-2.31 (7.2)	-2.80 (7.7)	-0.64 (6.81)	-2.01 (7.07)	-2.62 (7.46)	-1.14 (6.27)
Peak knee extension non paretic side (°)	-6.22 (5.79)	-7.14 (5.06)	-5.18 (5.1)	-5.75 (5.23)	-5.11 (5.14)	-5.12 (5.56)
Peak ankle dorsiflexion swing phase paretic side (°)	1.71 (6.92)	0.89 (6.43)	0.27 (6.32)	1.26 (7.28)	0.18 (8.67)	0.63 (7.39)
Peak ankle dorsiflexion swing phase non paretic side (°)	14.82 (6.36)	17.7 (9.22)	16.02 (7.44)	16.34 (6.17)	13.82 (6.09)	15.05 (6.89)
Peak ankle plantarflexion paretic side (°)	10.38 (7.88)	7.51 (7.81)	10.08 (7.99)	10.37 (7.79)	9.85 (9.54)	10.82 (8.6)
Peak ankle plantarflexion non paretic side (°)	9.61 (5.4)	7.01 (8.19)	10.22 (6.06)	11.26 (6.16)	9.92 (5.61)	10.73 (6.21)

*In Spont*: 4.83(1.18)s for Go, 2.98 (0.73)s for Turn and 4.23(1.02)s for Return;

*In Stand*: 4.56(1.01)s for Go, 3.16(0.84)s for Turn and 3.81(0.91)s for Return.

In Spont, sixteen participants turned towards the paretic side, 10 towards the non-paretic side and 3 changed turn direction within the 3 trials.

Median summed spasticity score was 4±3.6, median summed MRC score was 23±5.7, median pressure score on the sole of the foot was 1±0.5 and median proprioception score for the toe was 2±1.1 (both assessed with Nottingham Sensory Assessment). Eighteen subjects had claw toe in standing. Mean BBS score was 50.5±2.3, mean ABC score was 76.3±12.9. The median rate of falls was 1±0.7 and median fear of falling score was 2±3.2.

### Pearson’s correlation between Oriented gait and Turn performance and biomechanical parameters

#### Spont

Go: step length, %SP and %SSP on both sides and cadence and peak hip flexion on the paretic side were significantly negatively correlated with Go performance.

Turn: paretic step length and %SP and non-paretic %SSP were significantly negatively correlated with Turn performance.

Return: step length and %SSP on both limbs, non-paretic %SP and cadence were significantly negatively correlated with Return performance.

#### Stand

Go: step length, %SP and %SSP on both sides and cadence were significantly negatively correlated with Go performance.

Turn: %SP and %SSP on both sides and paretic step length and paretic peak knee extension were significantly negatively correlated with Turn performance.

Return: step length, %SP and %SSP on both sides and cadence were significantly negatively correlated with Return performance.

### Stepwise regression

#### In Spont

Step length on both sides and cadence were selected for Go, explaining 93% of the variance of Go performance.

Go performance=14.98−0.05paretic Step length−0.05Cadence−0.06non−paretic Step length

For Turn, non-paretic %SSPwas the only variable selected, explaining 27% of the variance of Turn performance.

➢ *Turn performance* = 6.8 − 0.08 non - paretic %SSPFor Return, step length on both sides and cadence were selected, explaining 82% of the variance of Return performance.

Return performance=13.7−0.05paretic Step length−0.05Cadence−0.06non−paretic Step length

#### In Stand

For Go, step length on both sides and cadence were selected, explaining 95% of the variance of Go performance.

➢Go performance=13.38−0.06paretic Step length−0.04Cadence−0.05non−paretic Step length

For Turn, non-paretic %SPand paretic %SSP were selected, explaining 56% of the variance of Turn performance.

Turn performance=5.23−0.39non−paretic%SP+0.32paretic%SSP

For Return, non-paretic %SP, cadence and step length on both sides were selected, explaining 87% of the variance of Return performance.

Return performance=13.1−0.09non−paretic%SP−0.04Cadence−0.04paretic Step length

Results of the stepwise analysis for both conditions are summarized in Table 3.

**Table 3:** Stepwise results for each sub-task and both conditions (Spont and Stand).

Spont		
Go	Turn	Return
Step length paretic side		Step length paretic side
step length non paretic side	%SSP non paretic side	step length non paretic side
cadence		cadence
Stand		
Go	Turn	Return
Step length paretic side	%SSP paretic side	Step length paretic side
step length non paretic side	%SP non paretic side	step length non paretic side
cadence		cadence
		%SP non paretic side

### Correlation between biomechanical parameters selected and clinical data

Table 4 presents the results of the correlation between the spatiotemporal and kinematic parameters selected in the stepwise analysis, and the clinical data.

**Table 4:** Correlation between Oriented gait and Turn performance explanatory spatiotemporal and kinematic parameters from the stepwise analysis and the clinical data.

	Spasticity	Claw toe	MRC	Foot sole pressure	Toe proprioception	BBS	ABC	Fall frequency	Fear of falling
Step length paretic side Go Spont	-0.04	0.03	0.11	0.20	0.06	0.46*	0.35	0.27	-0.33
Step length paretic side Return Spont	-0.18	-0.15	0.29	0.26	0.15	0.42*	0.22	0.24	-0.22
Step length non paretic side Go Spont	0.02	-0.02	0.25	0.17	0.19	0.43*	0.18	0.12	-0.41*
Step length non paretic side Return Spont	-0.08	-0.02	0.18	0.26	0.27	0.47*	0.13	0.03	-0.44*
Cadence Go Spont	0.27	0.01	-0.09	-0.12	-0.12	-0.04	0.31	0.32	-0.17
Cadence Return Spont	0.32	-0.07	-0.12	-0.16	-0.11	-0.06	0.36	0.27	-0.21
% SSP non paretic side Turn Spont	0.21	0.08	0.02	0.07	-0.07	0.04	0.36	-0.02	-0.08
Step length paretic side Go Stand	-0.11	0.03	0.16	0.16	0.03	0.44*	0.33	0.27	-0.30
Step length paretic side Return Stand	-0.10	0.10	0.12	0.13	0.05	0.26	0.26	0.42*	-0.26
Step length non paretic side Go Stand	0.00	-0.01	0.26	0.18	0.15	0.46*	0.25	0.14	-0.45*
Step length non paretic side Return Stand	-0.01	0.03	0.19	0.31	0.27	0.43*	0.20	0.15	-0.43*
Cadence Go Stand	0.21	-0.07	-0.11	-0.07	-0.08	0.01	0.30	0.27	-0.15
Cadence Return Stand	0.42*	-0.08	-0.14	-0.07	-0.06	-0.04	0.31	0.24	-0.18
% SSP paretic side Turn Stand	-0.20	-0.27	0.40*	0.11	0.03	0.36	0.22	0.38*	-0.40*
%SP non paretic side Turn Stand	-0.18	-0.28	0.39*	0.13	0.05	0.40*	0.25	0.43*	-0.36

MRC: Medical Research Council scale. BBS: Berg Balance Scale. ABC: Activities-specific Balance Confidence.

* significant correlation at p<0.05.

The BBS score was positively related to most parameters. MRC, fall frequency, fear of falling and MAS were only related to a few parameters. No correlations were found between the presence of claw toe, foot sole pressure score, toe proprioception score and ABC score and any biomechanical parameter.

## Discussion

To the best of our knowledge, the present study is the first to use 3D motion analysis to investigate spatiotemporal and kinematic parameters during Oriented gait and Turn sub-tasks of the TUG test in order to provide a deeper understanding of locomotor control in patients with stroke. The aim of this study was to determine the spatio-temporal and kinematic parameters which relate to performance in Oriented gait and Turn sub-tasks of the TUG in stroke patients. The results showed that in the spontaneous condition, step length on both sides and cadence best explained Go and Return performance, whereas percentage non-paretic SSP best explained Turn. In the standardized condition, the same parameters were selected in the stepwise analysis for Go and Return, and in addition, percentage non-paretic SP was selected in Turn and Return and percentage paretic SSP in Turn. Our hypothesis is partly confirmed since the percentage of single support phase was related to timed Turn performance but peak hip extension was not.

It is not surprising that step length and cadence explained performance in the walking sub-tasks since gait speed is the product of step length and cadence. Correlations have previously been found between total TUG time and gait speed [3]. Improvements in gait speed have also been shown to be more related to increased step length than other biomechanical variables after rehabilitation in stroke patients [39]. It is surprising that step width was not related to Oriented gait performance since this parameter is related to stability in stroke patients [40, 41]. However, increased step width increases the mechanical cost of gait in the frontal plane [42] which could explain the lack of association with forward progression in the walking sub-tasks of the TUG. Fear of falling was negatively correlated with step length on the non-paretic side, but not on the paretic side. Few studies have evaluated the relationship between fear of falling and step length in stroke. Park et al. showed that fear of falling was related to step cycle while walking but not to step length in only 12 stroke subjects, which contrasts with the present results [43]. However, many studies in elderly subjects have also demonstrated a relationship between fear of falling and decreased step length [44, 45, 46]. This is likely related to the fact that patients can more easily adapt non-paretic limb motion [47] in order to increase gait stability.

None of the kinematic parameters studied explained Oriented gait and Turn performance. This corroborates with a previous study which highlighted that kinematic parameters during conventional gait analysis were not predictive of the time to perform the entire TUG test [14]. Since increased gait speed is associated with increased hip extension and ankle dorsiflexion in patients with stroke [15, 48], it could be expected that TUG performance would be related to these parameters. The lack of association could be because of the short distance involved in the test. During the Go and Return sub-tasks, participants likely accelerated then decelerated before beginning the Turn task or the return to sit. So that these kinematic parameters are continuously adjusted during these sub-tasks and the net result is an absence of modification of the peaks.

Surprisingly, no correlations were found between MRC scores and the biomechanical parameters which were related to the Go and Return sub-tasks. Previous studies have shown that the best predictors of gait performance are strength of the paretic lower limb and balance in stroke patients [49]. Another study in our group showed correlations between time to perform the entire TUG and strength of the paretic limb [14]. This difference of results could be explained by the fact that in the present study we made correlation between the sum of the MRC score of the paretic lower limb and the performance at Oriented gait and Turn sub-tasks whereas in the previous one we performed distinct correlation between each muscle tested and the total TUG performance measured with a stopwatch. MAS score was related only to cadence during Return sub-task in Stand.

Percentage SSP appears to play an important role in the Turn sub-task although the limb (paretic or non-paretic) differed according to the condition. This corroboratesa previous study in our group showing that paretic %SSP assessed during conventional gait analysis is predictive of total TUG performance time in stroke patients [14]. In stroke patients, Ng and Hui-Chan (2005) found also a correlation between TUG performance and non-paretic stance time, and DeBujanda et al (2003) found a correlation with single support symmetry [3, 50]. Several studies have also shown a strong relationship between gait speed and single support time on the paretic limb in stroke patients [51, 52]. Gait speed being related to the time to perform the turn, it suggests that paretic limb loading and balance control on this limb are challenging during this sub-task. Moreover, turning requires a change of direction with deceleration of forward motion, rotation of the body and acceleration in a new direction [53]. It is a complex task for stroke patients who frequently evoke lacking balance during turning when they are asked about the circumstances of a fall [54]. Percentage of SSP on the paretic side is known to be closely linked with stability in stroke patients [51, 52]. Of all the clinical tests, the BBS score was related to the most biomechanical parameters in all the sub-tasks of the test. The correlation with %SP on the non-paretic side during the Turn in the standardized condition is probably due to the fact that, when one limb is in SSP the other is in SP,This indicates that the Turn is a good measure of balance capacity In the spontaneous condition, the participant could turn either towards the paretic or the non-paretic side which explain the lack of significance of %SP during the Turn. The MRC score was correlated with the biomechanical parameters which were related to the Turn. This suggests that more strength is required for this sub-task than for the walking sub-tasks. The stand-up and sit-down sub-tasks of the TUG may be affected by lower limb strength but were not assessed in this study.

To summarize, these results indicate that the walking sub-tasks of the TUG test which require a forward progression of the body are mainly affected by step length and cadence, while the turning sub-task of the TUG requires balance control which is related to the percentage of the gait cycle spent in stance phase. However, the percentage variance explained was high for the Go and Return sub-tasks (between 82% and 95%) and moderate for the Turn (27% and 56%) in both conditions. Both conditions (Spont and Stand) lead to the same explanatory parameters for walking sub-tasks (step length and cadence). In contrast, the Stand condition better explained the variance for the Turn sub-task, we therefore suggest that the standardized condition is more pertinent for the biomechanical assessment of Oriented gait and Turn sub-tasks of TUG performance. Confidence in carrying out activities (ABC score) was surprisingly not related to any of the biomechanical explanatory parameters. Oriented gait and Turn sub-tasks of TUG test appears thus more related to a global fear of falling than confidence to carry out specific activities.

### Limits

The sample of participants in the present study was a little younger (54.2±12.2 years) than other studies in the literature. However, it is unlikely that this would have influenced the results since Oriented gait and Turn sub-tasks of TUG performance was similar to that reported by Faria et al who found a time of 10.36s for the walking sub-tasks (summed) and 3.18s for the turning sub-task in patients with chronic stroke (mean age 59.12±2.28y) [29]. Similarly Botolfsen et al reported a time of 3.8 to 4.4 seconds for the walking sub-task and 3.8 to 4.2 seconds for the turning sub-task in older people with impaired mobility [55]. The mean score of BBS in our population (50.5±2.3) indicates good balance capacity [56], therefore the results of this study should be only be generalized to similar patients.

The differences found between the Spont and Stand conditions may be due to the fact that the Spont condition was always performed first. However, for the spontaneous condition to reflect spontaneous performance, it was essential for it to be carried out first. A similar methodology was used in another study [22].

## Conclusion

This study investigated spatiotemporal and kinematic parameters in Oriented gait and Turn sub-tasks of the TUG test in stroke patients. The results showed that step length and cadence explained most of the variance in the performance of the walking sub-tasks and, %SSP and %SP explained the turning sub-task. Balance capacity (assessed with BBS) and fear of falling were associated with the biomechanical parameters which explained performance in both the walking and the turning sub-tasks whereas spasticity, strength, sensation and proprioception were not, or only very slightly, related. It can thus be concluded that dynamic stability is the main capacity required to perform the walking and turning sub-tasks of the TUG. The results of the Spont and Stand conditions differed slightly, probably due to the different directions of the turn. More variance was explained in the standardized condition and therefore we suggest that this condition should be used to evaluate Oriented gait and Turn sub-tasks of TUG performance. This study demonstrated that biomechanical analysis of the Oriented gait and Turn sub-tasks of the TUG is useful to increase understanding of gait abnormalities. This is relevant for rehabilitation since the tasks evaluated by the TUG are highly functional and are carried out frequently throughout the day, however are rarely assessed using accurate tools. This analysis assesses balance capacity during gait, either for monitoring purposes or to evaluate the effects of treatment (rehabilitation, pharmaceutical or surgical). Moreover, the results of this assessment can be used to optimize rehabilitation, for example, to improve performance during the gait sub-tasks, rehabilitation should focus on increasing cadence and step length, whereas to improve the performance on the turning sub-task, balance capacity and particularly single support phase on the paretic side should be specifically trained.
